# Engineering a Quantitative Organ-on-a-Chip Platform for Myogenic Mechanobiology

**DOI:** 10.3390/bioengineering13030371

**Published:** 2026-03-23

**Authors:** Zepeng Zhou, Zhu Chen, Zhuojun Bai, Fengling Chen, Yujuan Huang, Yuan Guo

**Affiliations:** 1 Department of Cardiovascular Medicine, Zhuzhou Hospital Affiliated to Xiangya School of Medicine, Central South University, No. 116 South Changjiang Road, Zhuzhou 412007, China; 18834563351@163.com (Z.Z.); m13507332900@163.com (Z.B.); chenfengling8512@163.com (F.C.); yena_juan@163.com (Y.H.); 2 Institute for Future Sciences, University of South China, Changsha 410008, China; chenzhu220@163.com

**Keywords:** organ-on-a-chip, myogenic mechanobiology, cell maturation

## Abstract

Myogenic mechanobiology governs how mechanical cues regulate myocyte organization, alignment, and functional maturation; however, in vitro platforms that enable quantitative control and real-time readout of myogenic mechanical microenvironments remain limited. Here, we engineered a pneumatic-driven organ-on-a-chip platform integrating six parallel culture units and a bead-embedded flexible PDMS membrane to deliver cyclic mechanical strain and enable quantitative stress–strain mapping in cardiomyocytes and skeletal muscle cells. Finite element-guided optimization ensured effective membrane deformation, and the platform generated stable and tunable cyclic strain with a strong linear relationship between applied negative pressure (50–700 mbar) and membrane stress and strain. Plasma treatment combined with type I collagen coating restored myogenic cell adhesion and growth on PDMS to levels comparable to standard culture conditions. Under 13% cyclic strain, both cardiomyocytes and skeletal muscle cells exhibited pronounced and highly uniform alignment, with cellular polarity oriented perpendicular to the stretch axis. Moreover, cyclic loading significantly enhanced the expression of contractile maturation markers, including MYH7 in cardiomyocytes and MYH6 in skeletal muscle cells (all *p* < 0.05), whereas expression of the differentiation regulator MyoG remained unchanged, indicating that mechanical stimulation preferentially promotes structural organization and contractile maturation rather than lineage commitment. Collectively, this quantitatively programmable organ-on-a-chip represents a bioengineered microdevice for studying myogenic mechanobiology, revealing conserved mechanosensitive alignment and maturation responses across myogenic lineages and providing a versatile framework for biomedical engineering research, disease modeling, and mechanotherapeutic screening.

## 1. Introduction

Skeletal and cardiac muscles are highly mechanosensitive tissues whose development, remodeling, and function are critically regulated by mechanical cues [1]. During embryogenesis and postnatal maturation, myocytes are continuously exposed to cyclic stretch, contractile load, and matrix stiffness, which collectively govern their alignment, polarization, and acquisition of contractile phenotypes [2,3]. Dysregulation of these mechanobiological processes contributes to a wide spectrum of muscle pathologies, including cardiomyopathies, muscular dystrophies, and impaired regeneration [4,5,6]. Therefore, understanding how mechanical signals orchestrate muscle cell behavior is central to both fundamental myogenesis and translational muscle biology [7,8].

Myogenic mechanobiology refers to the conserved principles by which mechanical stimuli regulate structural organization and functional maturation across myogenic lineages, including cardiomyocytes and skeletal muscle cells. Accumulating evidence indicates that cyclic stretch can modulate cytoskeletal organization, sarcomere assembly, and contractile protein expression in both cell types [9,10]. However, most existing studies rely on conventional stretch systems or static culture platforms [11], which often lack precise control over the spatial and temporal profiles of mechanical cues and provide limited capability to quantify the actual forces experienced by cells. Consequently, this limitation has hindered systematic interrogation of myogenic mechanobiology under well-defined and reproducible biomechanical conditions [12].

Organ-on-a-chip technologies offer a promising avenue to overcome these limitations by integrating microengineering with cell biology to recapitulate key aspects of tissue microenvironments [13,14,15,16]. Recent muscle-on-a-chip platforms have enabled the generation of aligned myofibers and contractile tissues; yet many remain focused on specific muscle types or functional readouts, with insufficient emphasis on quantitative control and measurement of mechanical inputs [17,18]. In particular, the lack of systematic analysis and quantification of stress and strain distributions at the cell–substrate interface has constrained investigations into how externally applied mechanical stimuli regulate cellular mechanobiological responses [19].

Here, we designed to engineer a quantitatively programmable organ-on-a-chip platform to establish a unified in vitro framework for probing myogenic mechanobiology. By integrating pneumatic actuation with a fluorescent bead-embedded flexible polydimethylsiloxane (PDMS) membrane [20], the platform enables well-defined cyclic strain and real-time stress mapping. Using cardiomyocytes and skeletal muscle cells as representative myogenic models, we aimed to (i) optimize device geometry to achieve tunable mechanical stimulation, (ii) establish a biocompatible substrate that supports robust myogenic growth, and (iii) systematically evaluate how cyclic strain regulates alignment, polarization, and contractile maturation across distinct myogenic lineages. Through this work, we establish a quantitatively programmable myogenic organ-on-a-chip that defines a predictable mechanical microenvironment and provides a unified platform for dissecting conserved mechanobiological mechanisms across myocyte lineages.

## 2. Methods and Materials

### 2.1. Design of the Organ-on-a-Chip Device

The organ-on-a-chip device was designed to enable pneumatic actuation–driven stretching of a flexible membrane for controlled mechanical stimulation of cells. The overall architecture was constructed using Autodesk Fusion 360 version 2.0.19440 (Autodesk, Inc., San Francisco, CA, USA). Finite element analysis was conducted using COMSOL Multiphysics version 6.0 (COMSOL, Stockholm, Sweden) to optimize key structural parameters, including channel geometry, thereby enhancing device structural stability and improving the efficiency of mechanical signal transmission.

### 2.2. Finite Element Modeling

#### 2.2.1. Governing Equations

Finite element analysis (FEA) was performed to quantify the deformation behavior of the PDMS structure under pneumatic loading. The solid mechanics module was employed to solve the static equilibrium equation:

$$\nabla \cdot S\;+{\;F}_{V}\;=\;0$$

where S denotes the Cauchy stress tensor and $F_{V}$ represents the body force per unit volume.

PDMS was modeled as an isotropic hyperelastic material to capture its nonlinear elastic behavior under large deformation. Since the applied loading was quasi-static and did not involve high temperature, long-term loading, or time-dependent effects, viscoelasticity, creep, and thermal strain were not considered.

For elastic deformation, the constitutive relationship between stress and strain is defined using Hooke’s law in tensor form:

$$S_{\mathrm{el}}\;=\;C:\varepsilon_{\mathrm{el}}$$

$$S={\;S}_{\mathrm{el}}$$

where C = C(E,v) is the fourth-order elasticity tensor determined by the Young’s modulus E and Poisson’s ratio v of PDMS, and $\varepsilon_{\mathrm{el}}$ is the elastic strain tensor.

The strain–displacement relationship is expressed as:

$$\varepsilon \;=\;\frac{1}{2}[{(\nabla u)}^{T}+\nabla u]$$

where u represents the displacement vector field.

#### 2.2.2. Boundary Conditions

Upon application of negative pressure within the pneumatic channel, the adjacent PDMS sidewalls deform under the induced loading. The boundary traction condition is given by:

$$S\cdot n\;={\;F}_{A}$$

where n is the outward unit normal vector at the boundary surface and $F_{A}$ is the applied surface traction.

Because the applied air pressure is spatially uniform and always acts perpendicular to the loaded surface, the boundary traction simplifies to:

$$F_{A\;}=\;-\mathrm{pn}$$

where p is the applied pressure magnitude. The negative sign indicates that the pressure acts inward relative to the outward normal direction.

All other external surfaces were defined as fixed constraints or free boundaries according to the experimental configuration. The displacement field, strain distribution, and stress distribution within the PDMS structure were obtained by solving the stationary nonlinear problem.

### 2.3. Fabrication of the Organ-on-a-Chip Molds

Molds for the organ-on-a-chip were fabricated by computer numerical control (CNC) precision machining. Negative molds corresponding to each layer of the chip were designed, and computer-aided manufacturing (CAM) files were generated using Autodesk Fusion 360 version 2.0.19440. The CAM files were imported into a CNC system, and polymethyl methacrylate (PMMA) plates of appropriate dimensions were fixed onto the working platform for machining. After fabrication, surface burrs were removed using fine-grit sandpaper, yielding a final surface roughness of Ra = 0.150 ± 0.01 μm (*n* = 3). The molds were then thoroughly cleaned with absolute ethanol and air-dried.

### 2.4. Chip Fabrication and Assembly

The organ-on-a-chip devices were constructed using a layered fabrication and sequential bonding strategy, comprising a top medium reservoir, an upper cell culture chamber, a flexible membrane, and a lower pneumatic chamber. The fabrication of each layer was performed as follows. PDMS base and curing agent (Sylgard 184, Dow Corning, Midland, MI, USA) were mixed at a mass ratio of 10:1, thoroughly stirred, centrifuged at 3200 rpm for 1 min, and degassed under vacuum for 30 min. The mixture was poured into the corresponding molds and cured at 65 °C for 2 h. The cured PDMS layers were carefully peeled off, and inlet and outlet ports were punched.

For assembly, the upper layer and the flexible membrane were treated with oxygen plasma for 10 s and immediately bonded. Under microscopic observation, the membrane covering the side pneumatic channels was selectively removed to open the vacuum pathways. The structure was then successively bonded to the lower pneumatic layer and the top reservoir layer using the same plasma treatment and bonding procedure. Finally, the assembled chips were sterilized by autoclaving for 20 min and further exposed to UV irradiation for 1 h before use (Figure S1).

### 2.5. Fabrication of Fluorescent Bead-Embedded Flexible Membranes

Fluorescent microbeads (Haian Zhichuan Battery Material Technology Co., Ltd., Haian, China) (2 μm diameter, 5 mg) were uniformly dispersed in 1.00 ± 0.01 g of PDMS curing agent through sequential vortex mixing. After centrifugation, the supernatant was collected and weighed. PDMS base was then added at ten times the mass of the curing agent and thoroughly mixed to form a homogeneous prepolymer, followed by centrifugation for 1 min to remove air bubbles.

The prepolymer was dispensed onto a substrate and spin-coated at 1100 rpm for 30 s to form a uniform thin film, which was subsequently cured at 65 °C for 2 h. The resulting membrane was carefully peeled off, and its thickness was measured to be 97.33 ± 5.33 μm (*n* = 3), after which it was transferred onto a polyethylene terephthalate (PET) film and stored in the dark under sealed conditions until use.

### 2.6. Pneumatic Actuation Platform for Mechanical Stimulation

The pneumatic actuation system consisted of a benchtop pressure source, a microfluidic pressure controller, a vacuum pump, buffer reservoirs, switching valves, and gas tubing. Prior to experiments, all components were repeatedly wiped with 75% ethanol for cleaning and preliminary disinfection, then transferred into a biosafety cabinet and sterilized under UV light.

The organ-on-a-chip device was connected to the pressure source, pressure controller, and vacuum pump via tubing and quick connectors. During experiments, cyclic negative pressures with predefined amplitudes were applied under computer control. The chip was placed on an inverted fluorescence microscope (CKX53, OLYMPUS, Tokyo, Japan). The excitation light source was activated and an appropriate filter set was selected. The focus and exposure parameters were adjusted until a clear and stable fluorescence image was obtained, after which the imaging software was switched to recording mode to capture the displacement of fluorescent microbeads in real time during cyclic negative pressure actuation.

### 2.7. Measurement of Membrane Strain and Stress

Membrane strain and stress were quantified using traction force microscopy (TFM). Cyclic negative pressures ranging from −50 to −700 mbar were applied, and videos of bead motion during membrane deformation were acquired using an inverted fluorescence microscope (Supplementary Video S1).

The videos were converted into TIFF image sequences and batch-processed using custom ImageJ version 1.54j (National Institutes of Health, Bethesda, MD, USA) scripts for region of interest selection, noise reduction, bead signal enhancement, and background suppression. Particle image velocimetry (PIV) was performed using the PIVlab toolbox in MATLAB version R2024b (The MathWorks, Inc., Natick, MA, USA), with interrogation windows sequentially set to 128 (64), 64 (32), and 32 (16) pixels. Vector validation and interpolation were applied to obtain velocity fields.

The velocity data were further processed using custom MATLAB scripts to generate file formats compatible with the Fourier transform traction cytometry (FTTC) algorithm in ImageJ. Stress fields were calculated using the FTTC plugin in ImageJ [21], with the following parameters: pixel size = 2.29885 µm (determined from microscope calibration), Poisson’s ratio = 0.49 [22], Young’s modulus = 1,036,100 Pa (experimentally measured by uniaxial tensile testing of the prepared PDMS), and regularization parameter = 8 × 10^−11^ (selected according to established FTTC protocols). The material parameters are dependent on the PDMS formulation and experimental configuration. The resulting stress maps were further processed to remove outliers and missing values, and average stress and strain values were calculated for each pressure condition.

For visualization and quantitative analysis of the strain distribution, the strain maps generated from TFM were further processed. The strain field consisted of regularly spaced sampling points, each associated with a local strain value. Strain values were extracted row by row starting from the bottom of the map. Within each row, values were sequentially extracted from left to right, followed by the next row above, until all sampling points were included. The extracted strain profiles for each row were then fitted using a second-order polynomial to characterize the spatial strain distribution.

### 2.8. Cell Culture

Rat cardiomyoblast cell line H9c2 (CL-0089) and mouse skeletal muscle cell line C2C12 (CL-0044) were obtained from Wuhan Pricella Biotechnology Co., Ltd. (Wuhan, China). Cells were authenticated by short tandem repeat (STR) DNA profiling. Cells were cultured in high-glucose Dulbecco’s modified Eagle’s medium (DMEM; PM150210, Procell, Wuhan, China) supplemented with 10% fetal bovine serum (FBS; 164210, Procell, Wuhan, China) and 1% penicillin–streptomycin (PB180121, Procell, Wuhan, China). Cultures were maintained at 37 °C in a humidified atmosphere containing 5% CO_2_, with medium refreshed every 2 days.

When cells reached approximately 80–90% confluence, they were detached using 0.25% trypsin–EDTA solution (PB180226, Procell, Wuhan, China), collected by gentle centrifugation, resuspended in fresh complete medium, and passaged for subsequent experiments.

### 2.9. Preparation of PDMS-Coated Culture Dishes

To evaluate the influence of PDMS substrates on cell adhesion, PDMS-coated culture dishes were prepared. PDMS base and curing agent were mixed at a 10:1 mass ratio and degassed by low-speed centrifugation. Three small culture dishes were prepared: one dish was tilted using adhesive tape to form a sloped surface before adding PDMS prepolymer, another received PDMS prepolymer at the center to form a flat layer, and a third dish served as an untreated control.

All dishes were degassed in a vacuum chamber and cured at 65 °C for 2 h. After curing, they were immersed in 75% ethanol for at least 30 min and sterilized under UV irradiation in a biosafety cabinet for more than 1 h before use.

### 2.10. Type I Collagen Coating of PDMS Substrates

Rat tail tendon collagen type I (C8062, Solarbio, Beijing, China) was used to enhance cell adhesion on PDMS surfaces. Five PDMS-coated dishes were prepared and sterilized as described above. Collagen solutions at concentrations of 20, 40, 80, and 160 μg/mL were prepared in 0.36 g/L acetic acid. Each dish was incubated with 1.33 mL of the corresponding collagen solution at 37 °C and 5% CO_2_ for 1 h to ensure uniform coating.

After incubation, dishes were rinsed three times with PBS to remove unbound collagen. Cells were then seeded onto the coated substrates and cultured, with medium replaced every 48 h. Cell morphology and growth were monitored every 24 h, and cells were subjected to fluorescence staining at the end of culture.

### 2.11. Fluorescence Staining

For iFluor 594 wheat germ agglutinin (WGA; I3320, Solarbio, Beijing, China) staining, cells were fixed with 4% paraformaldehyde for 15 min, incubated with WGA working solution (5 μL WGA in 1 mL PBS) at room temperature in the dark for 30 min, rinsed with PBS, counterstained with 4′,6-diamidino-2-phenylindole (DAPI) for 8 min, mounted with antifade reagent, and imaged.

For live/dead staining, cells were incubated with Calcein AM and propidium iodide (PI; C2015S; Beyotime, Shanghai, China) working solution at 37 °C in the dark for 30 min, followed by fluorescence imaging.

For immunofluorescence staining, cells were fixed, permeabilized, and blocked according to standard protocols. Samples were then incubated with primary antibodies against MYH6 (1:100; A12964, ABclonal, Wuhan, China), MYH7 (1:100; A7564, ABclonal, Wuhan, China), or MyoG (1:500; ab1835, Abcam, Cambridge, UK) at 4 °C overnight. After washing, cells were incubated with Alexa Fluor 488– or Alexa Fluor 555–conjugated secondary antibodies (1:1000) for 60 min at room temperature in the dark. Nuclei were counterstained with DAPI. Fluorescence images were acquired using an inverted fluorescence microscope (CKX53, OLYMPUS, Tokyo, Japan), where the excitation light source was activated, the appropriate filter set was selected, and the focus and exposure parameters were adjusted until clear and stable images were obtained prior to image capture. The mean fluorescence intensity was quantified using ImageJ.

### 2.12. Statistical Analysis

All data are presented as mean ± standard error of the mean (SEM). Statistical analyses were performed using one-way analysis of variance (one-way ANOVA) in GraphPad Prism 10 (GraphPad Software, San Diego, CA, USA). Differences were considered statistically significant at *p* < 0.05.

## 3. Results

### 3.1. Design and Structural Optimization of an Organ-on-a-Chip for Myogenic Mechanobiology

**Figure 1:**
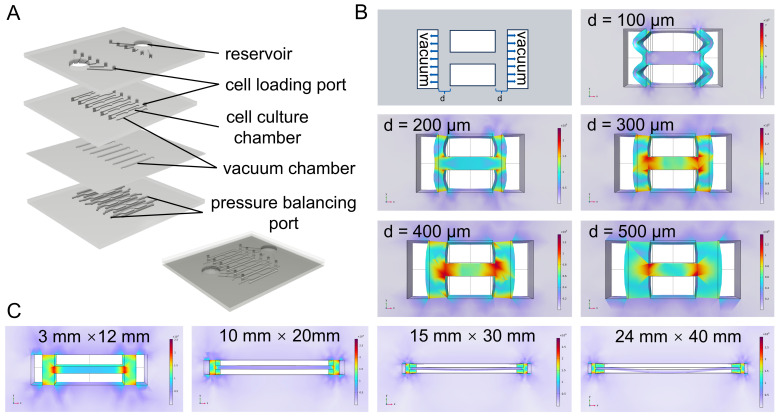
Design of the organ-on-a-chip and finite element analysis of key structural parameters. (**A**) Three-dimensional schematic of the organ-on-a-chip designed using Autodesk Fusion 360. (**B**) Finite element analysis of stress transmission across the PDMS layer separating the pneumatic actuation channel and the cell culture chamber, performed using COMSOL Multiphysics. The thickness of the PDMS layer was varied (*d* = 100, 200, 300, 400, and 500 μm). (**C**) Finite element analysis of membrane deformation under different cell culture areas (3 mm × 12 mm, 10 mm × 20 mm, 15 mm × 30 mm, and 24 mm × 40 mm), performed using COMSOL Multiphysics.

To recapitulate the biomechanical microenvironment governing myogenic differentiation and maturation, we developed a pneumatic-driven organ-on-a-chip platform capable of delivering well-defined cyclic strain to myogenic cells. The device integrates six parallel and identical culture units, enabling simultaneous multi-condition experiments and high-throughput mechanical modulation (Figure 1A and Figure S1).

Each unit consists of a 3-mm-wide cell culture channel and a 0.5-mm-wide vacuum channel, with the thickness (*d*) of the intervening PDMS layer critically determining strain transmission. COMSOL analysis revealed that at *d* = 100 μm, large bulk deformation occurred but the membrane was not effectively stretched, whereas at *d* = 500 μm, membrane deformation was markedly limited. An intermediate thickness of *d* = 400 μm produced substantial and uniform membrane strain (Figure 1B) and was therefore selected for subsequent myogenic mechanobiology studies.

Finite element simulations were further performed to evaluate the effect of culture area. These results showed that as the culture area increased, downward deflection of the flexible membrane became progressively more pronounced. At a culture area of 24 mm × 40 mm, excessive membrane deformation severely compromised both cell culture stability and mechanical signal transmission (Figure 1C). Accordingly, a culture area of 3 mm × 12 mm, comparable to that of a standard 96-well plate, was chosen as optimal.

### 3.2. Fabrication of a Fluorescent Bead-Embedded Flexible Membrane for Quantitative Myogenic Traction Analysis

To quantitatively characterize the strain and stress experienced by myogenic cells, fluorescent beads were embedded into the PDMS membrane for TFM analysis. Uniform bead dispersion was essential to ensure accurate mapping of mechanical fields.

**Figure 2:**
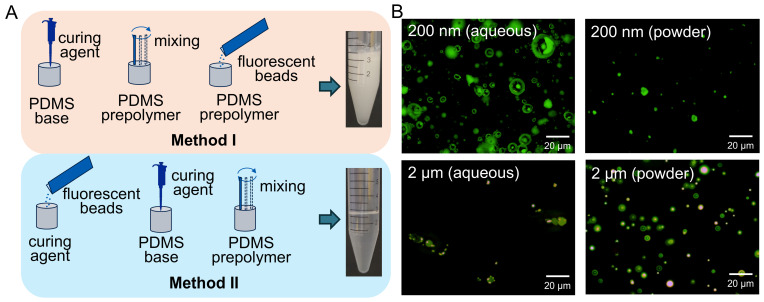
Fabrication and characterization of fluorescent bead–embedded flexible membranes. (**A**) Comparison of bead dispersion when fluorescent beads were added directly to the PDMS prepolymer versus pre-dispersed in the curing agent prior to mixing. (**B**) Representative fluorescence micrographs showing the distribution of embedded beads with different particle sizes within the flexible membrane (scale bar: 20 μm).

Direct mixing of beads into PDMS prepolymer led to severe aggregation, whereas pre-dispersion in the curing agent followed by mixing produced a transparent and homogeneous composite (Figure 2A). Bead size strongly influenced dispersion behavior: 200 nm beads exhibited frequent clustering, while 2 μm beads were uniformly distributed within the membrane. In addition, powder-form beads displayed superior dispersion stability compared with solution-based beads (Figure 2B). Accordingly, powder-form fluorescent beads with a diameter of 2 μm, pre-dispersed in the curing agent, were selected to fabricate bead-embedded membranes for subsequent quantitative mechanobiological analyses.

### 3.3. Quantitative Characterization of Controllable Cyclic Strain in the Myogenic Microenvironment

**Figure 3:**
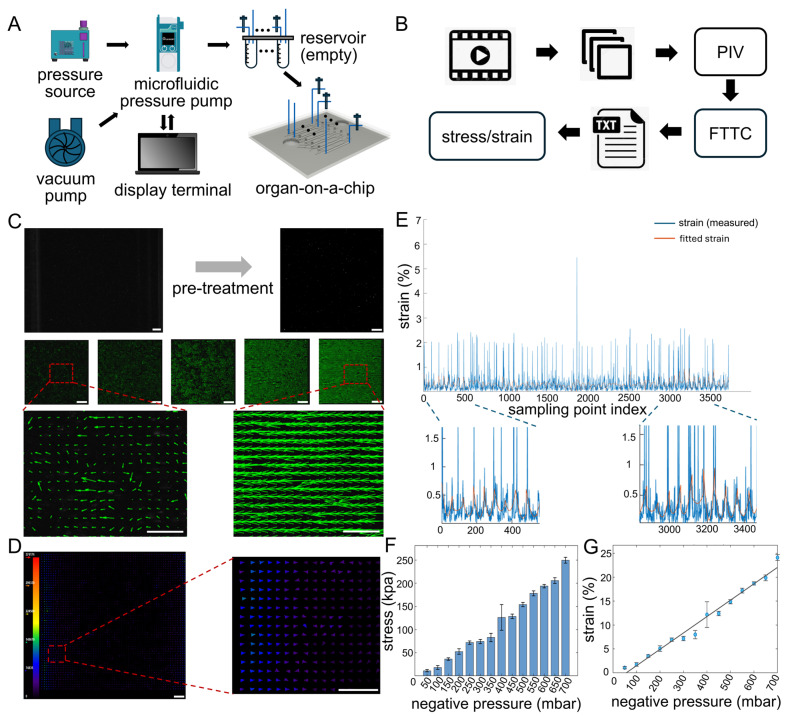
Quantitative measurement and analysis of membrane stress and strain under cyclic pneumatic actuation. (**A**) Schematic of the organ-on-a-chip integrated with the pneumatic actuation and imaging system. Arrows indicate the direction of gas flow or information exchange. (**B**) Workflow of traction force microscopy (TFM) for quantifying membrane deformation. Fluorescence videos of the stretched flexible membrane are acquired using an inverted fluorescence microscope and subsequently decomposed into sequential images at different time points. Particle image velocimetry (PIV) is then applied to the image series to extract the velocity field. Finally, stress and strain are computed from the velocity field in combination with the material properties of the flexible membrane using the Fourier transform traction cytometry (FTTC) algorithm. Arrows indicate the sequential steps of the workflow. (**C**) Representative images of preprocessing of fluorescent bead signals and displacement fields calculated by PIV. Arrow indicates the image pre-treatment process. (scale bar: 200 μm) (**D**) Representative stress map of the flexible membrane reconstructed using the FTTC algorithm. (scale bar: 200 μm) (**E**) Quantification of spatial strain distribution across the membrane. The blue line represents the measured strain values, and the orange line represents the fitted values. (**F**) Relationship between applied negative pressure and average membrane stress. (**G**) Relationship between applied negative pressure and average membrane strain. Data are presented as mean ± standard error of the mean (SEM) (*n* = 3).

The pneumatic actuation system, consisting of a pressure source, vacuum pump, microfluidic pressure controller, buffer reservoir, and organ-on-a-chip, enabled precise delivery of cyclic negative pressure to drive membrane stretching (Figure 3A). By integrating TFM, the system further allowed accurate quantification of stress during dynamic mechanical stretching (Figure 3B).

After image preprocessing, background noise was markedly reduced and bead fluorescence signals were enhanced. PIV revealed a periodic velocity field on the membrane surface that synchronized with the applied cyclic actuation (Figure 3C). The velocity fields were further converted into stress maps using the FTTC algorithm. The resulting stress distributions were heterogeneous, with stress levels increasing along the axis parallel to stretching and remaining relatively constant along the perpendicular axis (Figure 3D,E). Notably, despite this global heterogeneity, the stress field exhibited a relatively uniform distribution within localized central regions of the membrane (red dashed box, Figure S2).

When negative pressures ranging from 50 to 700 mbar were applied, both the average strain and stress increased monotonically, and the average strain exhibited a strong linear relationship with pressure (*R*^2^ = 0.980) (Figure 3F,G). These results confirm that the platform provides a stable, tunable, and quantitatively predictable mechanical microenvironment suitable for probing myogenic mechanobiology.

### 3.4. Surface Modification of PDMS to Support Myogenic Cell Adhesion and Growth

**Figure 4:**
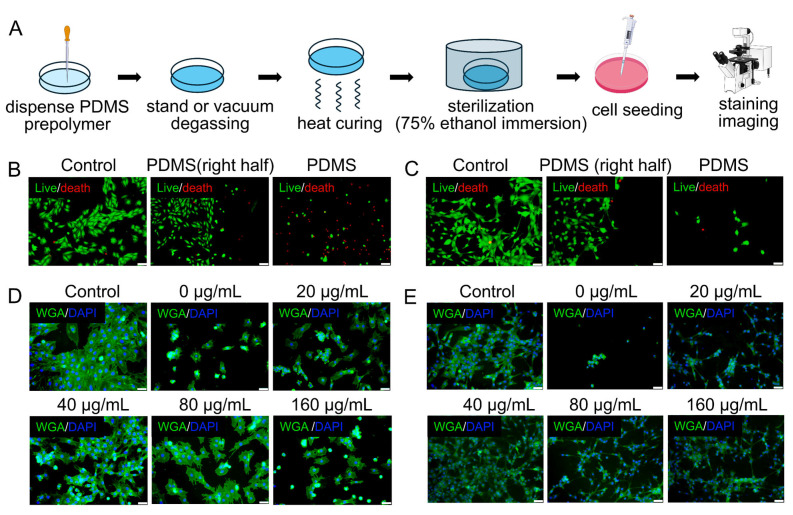
Effects of PDMS substrates on myogenic cell growth and their improvement by type I rat tail collagen coating. (**A**) Schematic of the experimental workflow for assessing the impact of PDMS on cell adhesion and growth. (**B**,**C**) Representative micrographs of cardiomyocytes and skeletal muscle cells cultured with or without PDMS substrates (scale bar: 100 μm). (**D**,**E**) Representative micrographs showing cardiomyocyte and skeletal muscle cell growth on PDMS surfaces coated with increasing concentrations of type I rat tail collagen (scale bar: 100 μm).

Both cardiomyocytes and skeletal muscle cells were used as representative myogenic models to evaluate the biocompatibility of the PDMS substrate (Figure 4A). On untreated PDMS surfaces, both cell types exhibited poor adhesion, abnormal spherical morphology, and reduced viability compared with standard culture controls (Figure 4B,C and Figure S3), indicating that native PDMS is suboptimal for sustaining myogenic growth.

Coating PDMS with type I rat-tail collagen significantly improved cell attachment and morphology in a concentration-dependent manner, with 40 μg/mL showing the most pronounced effect (Figure 4D,E), although growth remained inferior to that in standard controls.

**Figure 5:**
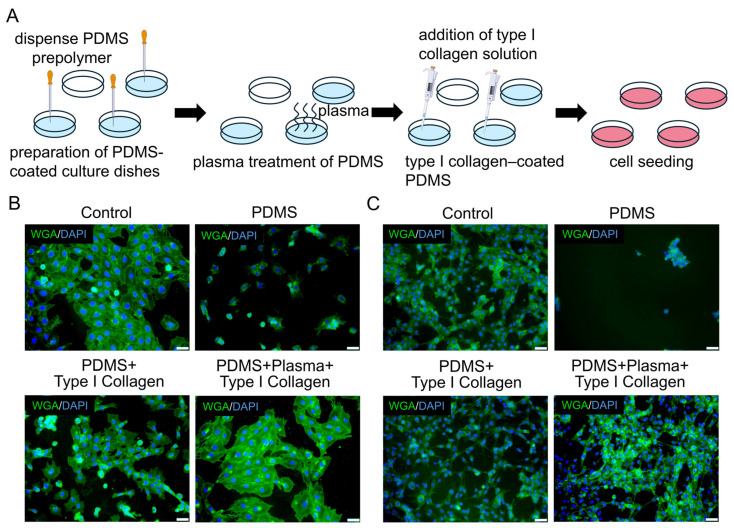
Enhancement of myogenic cell growth on PDMS substrates by different surface modification strategies. (**A**) Schematic illustration of the experimental workflow for PDMS surface modification. (**B**,**C**) Representative micrographs of cardiomyocytes and skeletal muscle cells cultured on PDMS surfaces subjected to different surface treatments (scale bar: 50 μm).

To further enhance substrate biocompatibility, PDMS surfaces were first treated with plasma to increase surface hydrophilicity, followed by collagen I coating (Figure 5A). This combined modification markedly restored cell adhesion, spreading, and cell density in both cardiomyocytes and skeletal muscle cells, achieving levels comparable to those observed in standard culture conditions (Figure 5B,C). Following this modification, cells cultured in the organ-on-a-chip exhibited stable morphology, uniform attachment, and robust growth, demonstrating that the substrate effectively supports cellular development as intended. Overall, this combined modification was therefore adopted to establish a robust and biocompatible substrate for long-term myogenic mechanobiology experiments on the chip.

### 3.5. Cyclic Mechanical Stimulation Coordinately Regulates Alignment of Myogenic Cells

**Figure 6:**
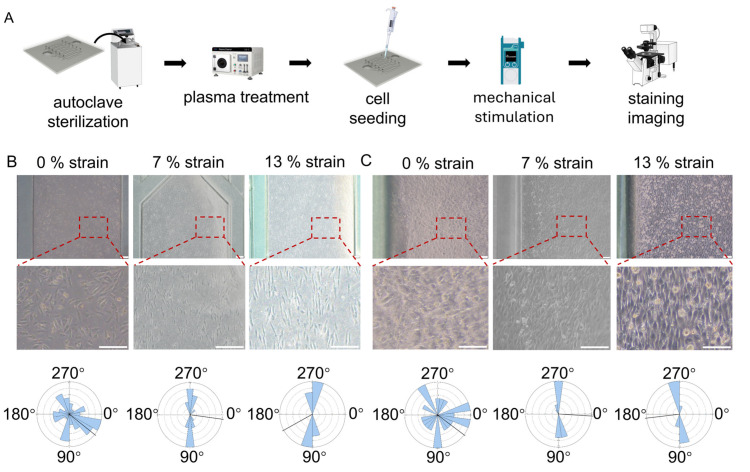
Effects of cyclic mechanical stimulation on the morphology and alignment of cardiomyocytes and skeletal muscle cells. (**A**) Schematic overview of the experimental workflow for applying cyclic mechanical stimulation to cardiomyocytes and skeletal muscle cells. (**B**,**C**) Representative micrographs and quantitative analysis of cell polarity for cardiomyocytes and skeletal muscle cells subjected to mechanical stimulation, respectively (scale bar: 200 μm in (**B**) and 100 μm in (**C**)).

To evaluate the mechanobiological responses of myogenic cells, cardiomyocytes and skeletal muscle cells were cultured on the chip and subjected to 0%, 7%, and 13% cyclic strain (Figure 6A, Supplementary Video S2). In both cell types, strains of 7% and 13% induced pronounced and highly uniform alignment, with cell polarity consistently oriented perpendicular to the stretching direction (Figure 6B,C). In contrast, cells cultured under static conditions displayed only local alignment within clusters and random global orientation (Figure 6B,C). These results indicate that cyclic strain acts as a dominant mechanical cue orchestrating large-scale architectural organization across distinct myogenic lineages.

### 3.6. Cyclic Strain Enhances the Contractile Maturation of Myogenic Phenotypes

**Figure 7:**
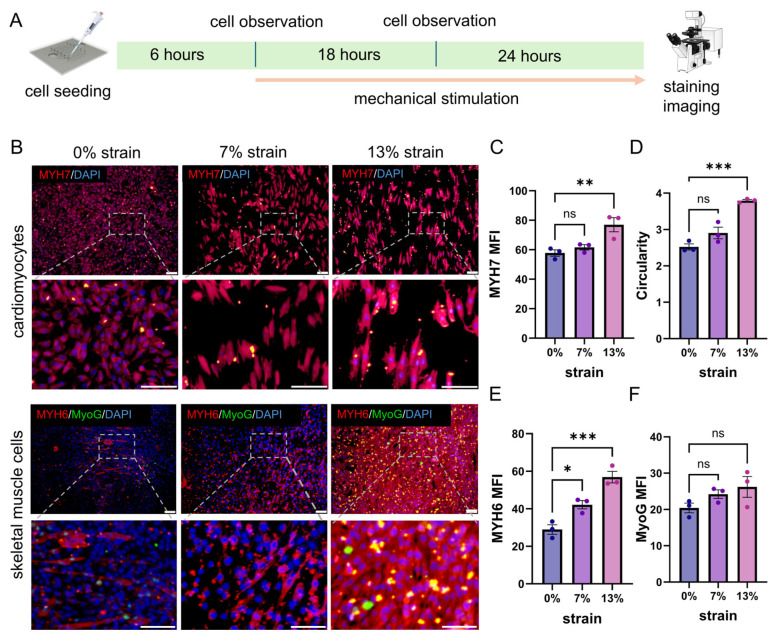
Effects of cyclic mechanical stimulation on functional protein expression in cardiomyocytes and skeletal muscle cells. (**A**) Schematic of the mechanical stimulation protocol applied to cardiomyocytes and skeletal muscle cells. (**B**) Representative immunofluorescence images showing functional protein expression in cardiomyocytes and skeletal muscle cells under different magnitudes of mechanical stimulation. In cardiomyocytes, MYH7 is shown (blue, DAPI; red, MYH7). In skeletal muscle cells, MYH6 and MyoG are shown (blue, DAPI; red, MYH6; green, MyoG) (scale bar: 100 μm). (**C**) Quantitative analysis of MYH7 expression in cardiomyocytes based on mean fluorescence intensity (MFI). (**D**) Statistical analysis of cardiomyocyte circularity under different mechanical stimulation conditions. (**E**,**F**) Quantitative analysis of MYH6 and MyoG expression in skeletal muscle cells based on MFI, respectively. Data are presented as mean ± standard error of the mean (SEM) (*n* = 3). Statistical significance was assessed using one-way analysis of variance (ANOVA). ns, not significant; * *p* < 0.05, ** *p* < 0.01, *** *p* < 0.001.

Cyclic mechanical stimulation was applied according to a defined time-course protocol, initiated 6 h after cell seeding and maintained for up to 48 h (Figure 7A). Under this dynamic loading regimen, cyclic strain markedly promoted the functional maturation of myogenic cells. In cardiomyocytes, MYH7 fluorescence intensity was significantly increased under 13% strain (76.99 ± 4.80) compared with static controls at 0% strain (57.77 ± 2.17, *p* = 0.0098). Consistently, cell elongation, quantified by the ratio of the major to minor cell axis (cell circularity), was markedly enhanced under 13% strain (3.794 ± 0.029) relative to the 0% strain condition (2.522 ± 0.085, *p* = 0.0003, Figure 7B–D).

Similarly, in skeletal muscle cells, expression of the contractile protein MYH6 increased progressively with increasing strain magnitude, from 28.94 ± 2.54 at 0% strain to 42.20 ± 2.24 at 7% strain and 56.91 ± 3.06 at 13% strain (all *p* < 0.05, Figure 7E).

In contrast, expression of the differentiation regulator MyoG in skeletal muscle cells did not show significant differences among the tested conditions (Figure 7F), suggesting that cyclic strain primarily facilitates structural reorganization and contractile phenotype maturation rather than serving as a sole trigger for lineage commitment.

Collectively, these findings demonstrate that the organ-on-a-chip-generated mechanical microenvironment exerts a conserved mechanobiological effect across myogenic cell types, driving coordinated alignment and enhancement of contractile maturation.

## 4. Discussion

In this study, we developed a quantitatively programmable organ-on-a-chip platform to interrogate myogenic mechanobiology by integrating tunable cyclic strain, real-time stress mapping, and parallelized culture. Through rational device design and surface engineering, we established a controllable biomechanical microenvironment that supports robust culture of both cardiomyocytes and skeletal muscle cells. Using these two representative myogenic lineages, we demonstrate that cyclic mechanical stimulation elicits conserved structural and functional responses, providing a unified experimental framework for investigating mechanoregulation across myocyte lineages.

A key advancement of this platform is the precise control and quantitative characterization of the mechanical microenvironment. Finite element modeling guided optimization of geometric parameters governing strain transmission, yielding stable and spatially homogeneous deformation. The linear relationship between applied negative pressure and membrane stress and strain confirms the predictability and reproducibility of mechanical stimulation. In contrast to conventional stretch systems that report nominal strain without resolving spatial heterogeneity [23,24], integration of traction force microscopy enables direct mapping of cell–substrate stress distributions, thereby establishing a quantitative basis for mechanical dose–response analysis in myogenic mechanobiology.

Using this well-defined mechanical framework, we identified a conserved alignment response across distinct myogenic lineages. Under cyclic strain, both cardiomyocytes and skeletal muscle cells exhibited pronounced alignment perpendicular to the stretching axis, whereas static cultures remained disorganized. This behavior is consistent with the classical strain-avoidance response, whereby cyclic deformation drives force-dependent reorganization of the actin cytoskeleton and focal adhesions to minimize mechanical load [25,26]. Importantly, the convergence of alignment responses across muscle types suggests that shared integrin-mediated mechanosensing and actomyosin contractility pathways underlie myogenic responses to cyclic strain, independent of lineage-specific differentiation programs [9].

Beyond structural reorganization, cyclic strain selectively promoted contractile maturation in both lineages, as reflected by increased expression of lineage-appropriate myosin heavy chain isoforms (MYH7 in cardiomyocytes and MYH6 in skeletal muscle cells) [27,28]. In contrast, expression of the early myogenic differentiation regulator MyoG in skeletal muscle cells remained unchanged, indicating that mechanical stimulation enhances post-commitment maturation rather than driving lineage specification. This dissociation underscores the stage-specific role of mechanical cues in myogenesis, supporting a model in which cyclic strain refines sarcomeric organization and functional capacity after the myogenic program has been established.

Mechanistically, such selective enhancement of maturation is likely mediated by strain-induced modulation of cytoskeletal tension and sarcomeric assembly rather than activation of upstream fate-determining transcription factors [29]. Mechanical loading has been shown to influence excitation–contraction coupling and contractile architecture in muscle cells [30], thereby promoting functional refinement without altering lineage identity. Our findings are consistent with this framework and suggest that cyclic mechanical cues primarily act on downstream structural modules of the myogenic program.

From a bioengineering perspective, our results highlight the importance of substrate biocompatibility in mechanobiology-on-chip systems. While PDMS is widely used in microfluidic platforms, its native surface was insufficient to support myogenic adhesion and growth [31,32]. Previous studies have demonstrated that plasma treatment, chemical modification, and collagen coating can effectively enhance cell adhesion on PDMS surfaces. However, plasma treatment alone is limited by hydrophobic recovery, whereas chemical modification, despite its improved stability, involves relatively complex procedures [33,34].

It has been reported that plasma activation combined with collagen coating can improve surface stability [35]. Consistent with these findings, which were primarily obtained under static culture conditions, we applied this combined strategy in a dynamic stretching system. This approach enabled stable cell attachment and morphological maintenance while maintaining procedural simplicity and effective sterilization. It thereby provided a reliable interface for long-term mechanical stimulation and minimized interference arising from suboptimal cell–material interactions.

Compared with existing muscle-on-a-chip platforms that predominantly focus on generating contractile tissues or measuring force output [36], our system is uniquely suited to interrogate input–output relationships in myogenic mechanobiology by linking defined mechanical stimuli to cellular structural and molecular responses. The six-unit parallelized design further enables multi-condition testing within a single device, enhancing experimental throughput and reproducibility. This feature is particularly advantageous for systematically exploring the effects of varying strain amplitudes, frequencies, or waveforms, as well as for evaluating pharmacological or biomaterial interventions under dynamically controlled mechanical conditions [37]. This capability positions the platform not only as an engineering tool, but also as a discovery engine for identifying threshold-dependent and non-linear mechanotransduction regimes in myocytes.

Several limitations should be acknowledged. The current platform models a simplified two-dimensional cell–substrate interface and does not recapitulate the three-dimensional architecture and multicellular complexity of native muscle tissue. In addition, while substrate stress mapping provides insight into the external mechanical environment, intracellular force transmission and downstream mechanotransduction signaling were not directly assessed. Future integration of three-dimensional matrices and real-time testing of mechanosensitive signaling would further enhance the physiological relevance and mechanistic resolution of the system.

Collectively, our results establish a versatile organ-on-a-chip framework that bridges microengineering and muscle biology, enabling quantitative dissection of how mechanical cues orchestrate myogenic organization and maturation. By revealing conserved mechanobiological responses across cardiac and skeletal muscle cells, this work supports the emergence of myogenic mechanobiology as a unifying paradigm for understanding muscle mechanoregulation in both health and disease in future.

## 5. Conclusions

In conclusion, a quantitatively programmable organ-on-a-chip platform is established to recapitulate key biomechanical features of the myogenic microenvironment and enable systematic interrogation of myogenic mechanobiology. Integration of optimized device architecture, bead-embedded flexible membranes, and biocompatible surface engineering allows stable and predictable cyclic strain delivery with direct quantitative mapping of stress and strain at the cell–substrate interface. Studies in cardiomyocytes and skeletal muscle cells demonstrate that cyclic mechanical loading elicits conserved mechanobiological responses, characterized by coordinated cellular alignment and enhanced contractile maturation, supporting a unified mechanoregulation program across myogenic lineages. Collectively, this myogenic mechanobiology-on-chip framework provides a robust in vitro platform for quantitative studies of muscle mechanoregulation with broad translational potential in disease modeling, mechanotherapeutic development, and drug screening in mechanically active muscle microenvironments.

## Data Availability

The original contributions presented in this study are included in the article/Supplementary Material. Further inquiries can be directed to the corresponding author.

## Supplementary Materials

The following supporting information can be downloaded at: https://www.mdpi.com/article/10.3390/bioengineering13030371/s1. Figure S1. Fabrication procedure and optical image of the organ-on-a-chip. Figure S2. Analysis of stress uniformity in the flexible substrate membrane under mechanical loading. Figure S3. Growth of cardiomyocytes and skeletal muscle cells cultured on PDMS. Supplementary Video S1. Fluorescent bead displacement of the flexible membrane in the organ-on-a-chip under cyclic stretching. Supplementary Video S2. Cardiomyocytes in the organ-on-a-chip under cyclic stretching.
