# Supplementary material for: Engineering a Quantitative Organ-on-a-Chip Platform for Myogenic Mechanobiology
Source: Bioengineering (Basel). 2026 Mar 23;13(3):371. doi: 10.3390/bioengineering13030371 (PMC13024678; doi:10.3390/bioengineering13030371)
Supplement: Supplementary file 1 [file bioengineering-13-00371-s001.zip › Supplementary Information.pdf]

Supplementary material

Engineering a Quantitative Organ-on-a-Chip Platform for Myogenic Mechanobiology

Zepeng Zhou[1, †], Zhu Chen[2, †], Zhuojun Bai[1], Fengling Chen[1], Yujuan Huang[1], Yuan Guo[1, *]

[1] Department of Cardiovascular Medicine, Zhuzhou Hospital Affiliated to Xiangya School of Medicine, Central South University, No. 116 South Changjiang Road, Zhuzhou 412007, China; 18834563351@163.com (Z.Z.); m13507332900@163.com (Z.B.); chenfengling8512@163.com (F.C.); yena_juan@163.com (Y.H.)

[2] Institute for Future Sciences, University of South China, Changsha 410008, China; chenzhu220@163.com

***Corresponding Author**: *Yuan Guo, Department of Cardiovascular Medicine, Zhuzhou Hospital Affiliated to Xiangya School of Medicine, Central South University, No. 116 South Changjiang Road, Zhuzhou 412007, Hunan, China. E-mail: guoyuan8141@csu.edu.cn*.

[†] These authors contributed equally to this work.

Figure S1

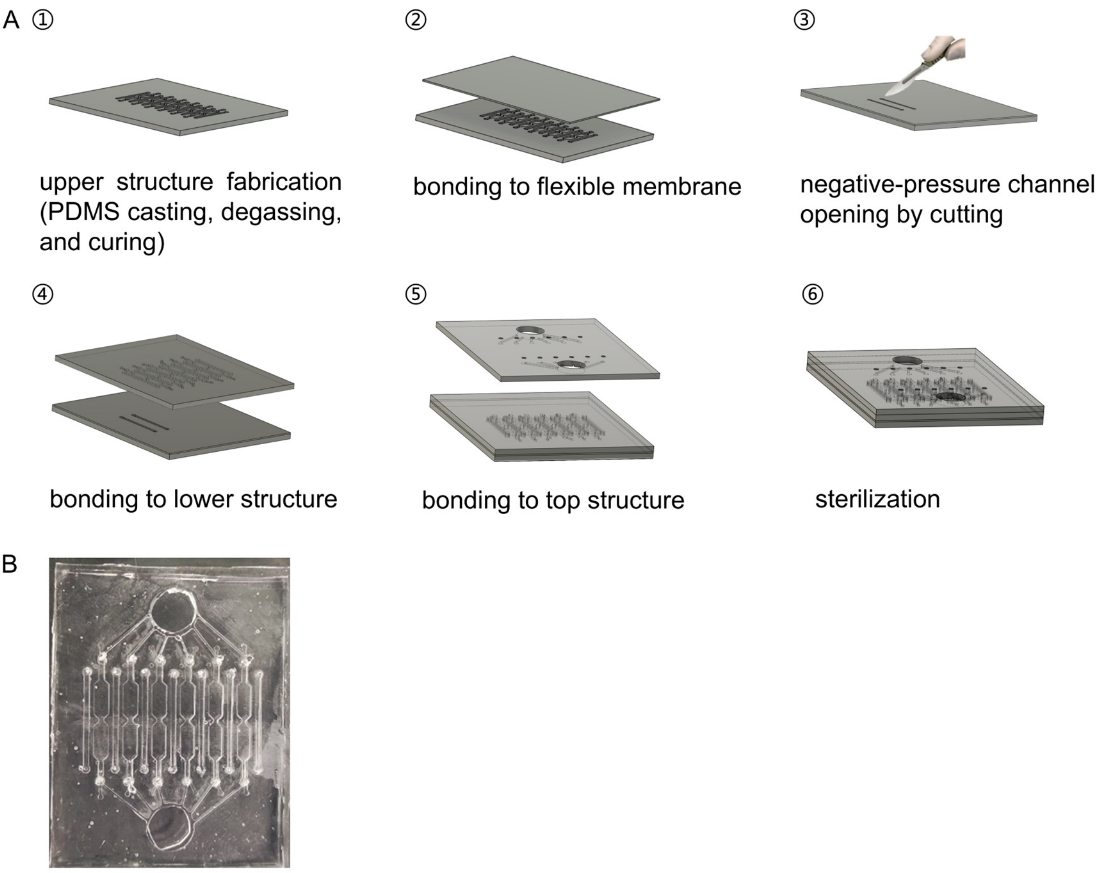

Figure S1. Fabrication procedure and optical image of the organ-on-a-chip.

(A) Device fabrication workflow: first, individual layers were fabricated by PDMS casting, degassing, and thermal curing. The upper layer was then bonded to a flexible membrane via oxygen plasma treatment. Subsequently, the flexible membrane over the negative-pressure channel was carefully removed using a scalpel. The resulting assembly was bonded to the bottom layer, followed by bonding to the top layer. Finally, the assembled device was sterilized by high-pressure steam autoclaving. (B) Optical image of the fabricated organ-on-a-chip.

Figure S2

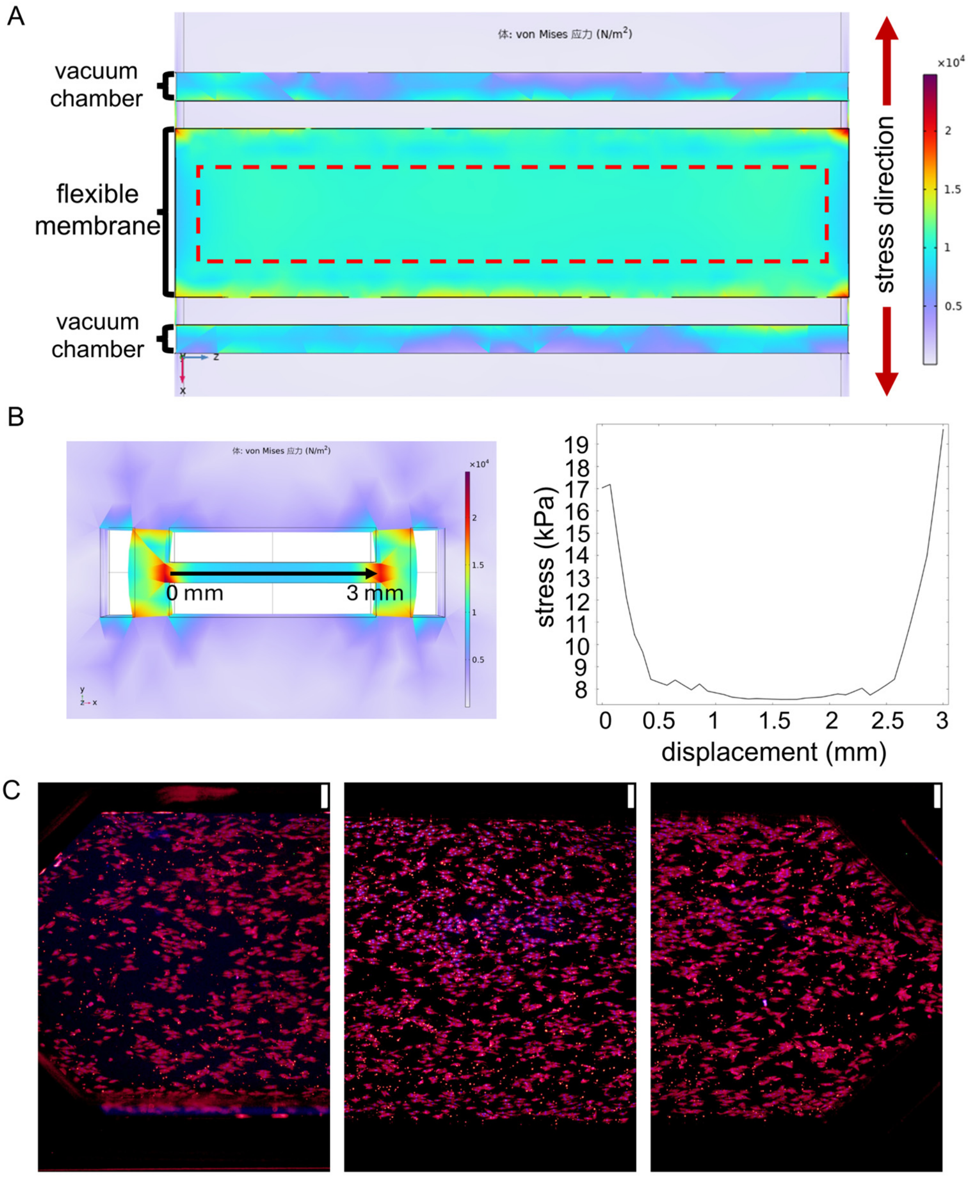

Figure S2. Analysis of stress uniformity in the flexible substrate membrane under mechanical loading.

(A) Finite element analysis of the stress distribution in the flexible substrate membrane subjected to mechanical loading. (B) Displacement–stress profile extracted along the black line from left to right, plotted as a line graph. (C) Representative images showing cardiomyocyte growth over a large area under mechanical stretching, including cell morphology, spatial distribution, and cell density. Scale bar = 200 μm.

Figure S3

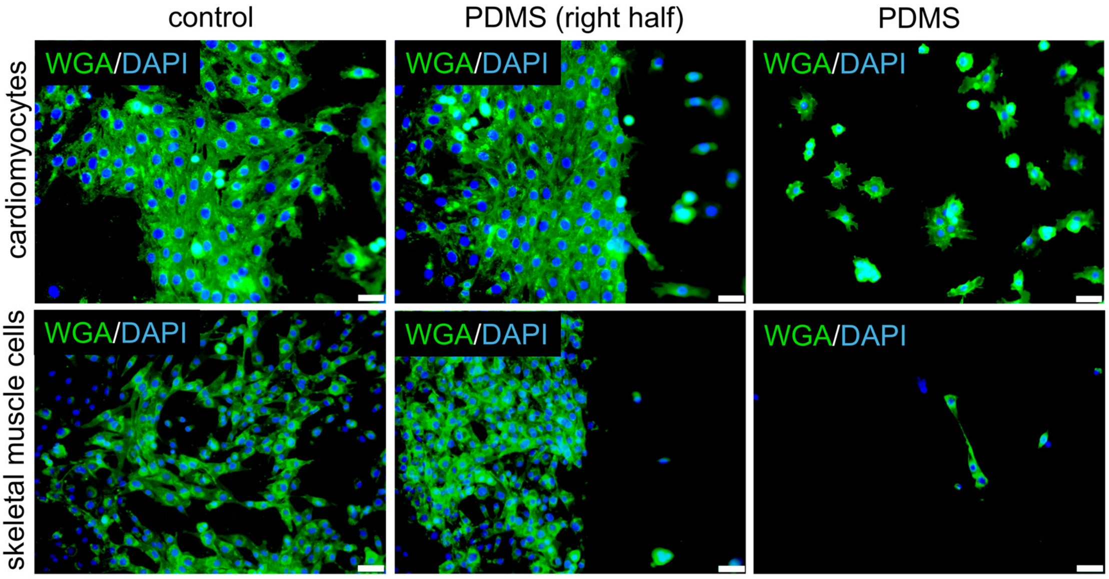

Figure S3. Growth of cardiomyocytes and skeletal muscle cells cultured on PDMS. Cell membranes are shown in green and nuclei are shown in blue. Scale bar = 50 µm.
